# Supplementary material for: Alternate patterns of temperature variation bring about very different disease outcomes at different mean temperatures
Source: eLife. 2022 Feb 15;11:e72861. doi: 10.7554/eLife.72861 (PMC8846586; doi:10.7554/eLife.72861)
Supplement: Supplementary file 1. [file elife-72861-supp1.docx]

Table S1. Priors for each of the parameters in the Beta Function for each of the different (*j*) temperature regimes (constant, fluctuating, heatwave) and life history traits (infection rate, spore burden, reproductive output), which were all drawn from the uniform distribution with specified limits. Priors for the minimum, optimal and maximum temperature satisfy the necessary condition ***T_min_***≤***T_opt_***≤***T_max_*** and were informed by previous work (Kirk et al., 2018, 2019). Priors for the scaling parameter ***F_m_*** were restricted to be positive and less than ten on the log10 scale for both spore burden and host reproductive output and between 0 and 1 for infection rates. A Poisson likelihood was used for the observed reproductive output of the host and spore burden and the rate parameter 𝜆 was modelled as a function of temperature with a log link function, with different parameter values for each of the three temperature regimes. For infection rates we used a similar approach but using a binomial likelihood where the probability *p* was estimated from the Beta Function constrained so that 0 ≤ *p* ≤ 1 and *N* was the number of *Daphnia* in each temperature regime.

| **Parameter** | **Infection rate**  **(min, max)** | **Spore burden**  **(min, max)** | **Host reproductive output**  **(min, max)** |
| --- | --- | --- | --- |
| ***F_m_*** [*j*] | 0,1 | 0,10 | 0,10 |
| ***T_min_*** [*j*] | 5, 15 | 5,14 | 0,14 |
| ***T_opt_*** [*j*] | ***T_min_*** [*j*] + 0, ***T_min_*** [*j*] + 20 | ***T_min_*** [*j*] + 0, ***T_min_*** [*j*] + 10 | ***T_min_*** [*j*] + 0, ***T_min_*** [*j*] + 25 |
| ***T_max_*** [*j*] | ***T_opt_*** [*j*] + 0, 35 | ***T_opt_*** [*j*] + 0, 30 | ***T_opt_*** [*j*] + 0, 40 |
