# Supplementary material for: Alternate patterns of temperature variation bring about very different disease outcomes at different mean temperatures
Source: eLife. 2022 Feb 15;11:e72861. doi: 10.7554/eLife.72861 (PMC8846586; doi:10.7554/eLife.72861)
Supplement: Supplementary file 2. [file elife-72861-supp2.docx]

Table S2. Estimates of the parameters of the Beta Function for infection rate over the different temperature regimes. Provided are the mean thermal minimum (***T_min_***), maximum (***T_max_***) and thermal optimum (***T_opt_***), as well as the maximum infection rate (***F_m_***) with 95% confidence interval (lower CI, 2.5% and upper 97.5%). The sample sizes for these estimates were respectively 82, 85 and 63 for constant, fluctuating and heat wave regimes.

| **Variable** | **Temperature regime** | **Infection status** | **Mean** | **CI 2.5%** | **CI 97.5%** |
| --- | --- | --- | --- | --- | --- |
| ***F_m_*** | constant | exposed | 0.96 | 0.87 | 1.00 |
| ***F_m_*** | fluctuating | exposed | 0.94 | 0.84 | 1.00 |
| ***F_m_*** | heat wave | exposed | 0.95 | 0.85 | 1.00 |
| ***T_max_*** | constant | exposed | 30.23 | 27.98 | 34.04 |
| ***T_max_*** | fluctuating | exposed | 24.98 | 24.32 | 26.41 |
| ***T_max_*** | heat wave | exposed | 29.49 | 24.96 | 34.56 |
| ***T_min_*** | constant | exposed | 9.86 | 6.39 | 10.70 |
| ***T_min_*** | fluctuating | exposed | 10.75 | 6.10 | 12.33 |
| ***T_min_*** | heat wave | exposed | 10.88 | 5.42 | 13.79 |
| ***T_opt_*** | constant | exposed | 19.72 | 18.13 | 21.27 |
| ***T_opt_*** | fluctuating | exposed | 19.06 | 17.74 | 20.15 |
| ***T_opt_*** | heat wave | exposed | 19.23 | 17.49 | 20.95 |
