# Supplementary material for: Alternate patterns of temperature variation bring about very different disease outcomes at different mean temperatures
Source: eLife. 2022 Feb 15;11:e72861. doi: 10.7554/eLife.72861 (PMC8846586; doi:10.7554/eLife.72861)
Supplement: Supplementary file 3. [file elife-72861-supp3.docx]

Table S3. Estimates of the parameters of the Beta Function for parasite burden over the different temperature regimes. Provided are the mean thermal minimum (***T_min_***), maximum (***T_max_***) and thermal optimum (***T_opt_***), as well as the estimated number of spores at the thermal optimum (***F_m_***) with their 95% confidence interval (lower CI, 2.5% and upper 97.5%). The sample sizes for these estimates were respectively 51, 46 and 46 for constant, fluctuating and heat wave regimes.

| **Variable** | **Temperature regime** | **Infection status** | **Mean** | **CI 2.5%** | **CI 97.5%** |
| --- | --- | --- | --- | --- | --- |
| ***F_m_*** | constant | exposed | 489.78 | 478.63 | 501.19 |
| ***F_m_*** | fluctuating | exposed | 467.74 | 446.68 | 478.63 |
| ***F_m_*** | heat wave | exposed | 794.33 | 758.58 | 831.76 |
| ***T_max_*** | constant | exposed | 27.70 | 27.45 | 27.95 |
| ***T_max_*** | fluctuating | exposed | 24.80 | 24.52 | 25.07 |
| ***T_max_*** | heat wave | exposed | 28.73 | 28.17 | 29.37 |
| ***T_min_*** | constant | exposed | 11.49 | 10.75 | 12.11 |
| ***T_min_*** | fluctuating | exposed | 11.48 | 10.18 | 12.32 |
| ***T_min_*** | heat wave | exposed | 13.91 | 13.90 | 13.91 |
| ***T_opt_*** | constant | exposed | 19.44 | 19.34 | 19.54 |
| ***T_opt_*** | fluctuating | exposed | 19.30 | 19.23 | 19.39 |
| ***T_opt_*** | heat wave | exposed | 15.76 | 15.53 | 15.97 |
