# Supplementary material for: Alternate patterns of temperature variation bring about very different disease outcomes at different mean temperatures
Source: eLife. 2022 Feb 15;11:e72861. doi: 10.7554/eLife.72861 (PMC8846586; doi:10.7554/eLife.72861)
Supplement: Supplementary file 4. [file elife-72861-supp4.docx]

Table S4. Estimates of the parameters of the Beta Function for the reproductive output of the host over the different temperature regimes. Provided are the mean thermal minimum (***T_min_***), maximum (***T_max_***) and thermal optimum (***T_opt_***), as well as the estimate for the highest reproductive output (***F_m_***) with their and 95% confidence interval (lower CI, 2.5% and upper 97.5%). The sample size for these estimates were 81 and 81 for constant, 85 and 87 for fluctuating, and 64 and 61 for heat wave regimes for infected and uninfected individuals respectively.

| **Variable** | **Temperature regime** | **Infection status** | **Mean** | **CI 2.5%** | **CI 97.5%** |
| --- | --- | --- | --- | --- | --- |
| ***F_m_*** | constant | exposed | 101.39 | 97.44 | 105.20 |
| ***F_m_*** | constant | unexposed | 109.65 | 105.74 | 113.76 |
| ***F_m_*** | fluctuating | exposed | 81.28 | 78.71 | 84.14 |
| ***F_m_*** | fluctuating | unexposed | 106.41 | 102.85 | 110.15 |
| ***F_m_*** | heat wave | exposed | 92.90 | 89.79 | 96.16 |
| ***F_m_*** | heat wave | unexposed | 101.39 | 97.37 | 105.68 |
| ***T_max_*** | constant | exposed | 36.22 | 35.37 | 37.16 |
| ***T_max_*** | constant | unexposed | 37.58 | 36.73 | 38.51 |
| ***T_max_*** | fluctuating | exposed | 38.90 | 36.54 | 39.97 |
| ***T_max_*** | fluctuating | unexposed | 31.06 | 30.49 | 31.72 |
| ***T_max_*** | heat wave | exposed | 39.46 | 38.11 | 39.99 |
| ***T_max_*** | heat wave | unexposed | 34.68 | 30.79 | 39.38 |
| ***T_min_*** | constant | exposed | 10.50 | 10.22 | 10.68 |
| ***T_min_*** | constant | unexposed | 10.59 | 10.34 | 10.70 |
| ***T_min_*** | fluctuating | exposed | 6.66 | 3.57 | 8.45 |
| ***T_min_*** | fluctuating | unexposed | 0.63 | 0.02 | 2.18 |
| ***T_min_*** | heat wave | exposed | 13.09 | 12.72 | 13.36 |
| ***T_min_*** | heat wave | unexposed | 7.00 | 0.95 | 10.57 |
| ***T_opt_*** | constant | exposed | 19.61 | 19.43 | 19.80 |
| ***T_opt_*** | constant | unexposed | 20.22 | 20.05 | 20.39 |
| ***T_opt_*** | fluctuating | exposed | 20.50 | 20.12 | 20.89 |
| ***T_opt_*** | fluctuating | unexposed | 20.26 | 20.12 | 20.40 |
| ***T_opt_*** | heat wave | exposed | 18.52 | 18.23 | 18.83 |
| ***T_opt_*** | heat wave | unexposed | 21.16 | 20.74 | 21.69 |
