# Supplementary material for: Alternate patterns of temperature variation bring about very different disease outcomes at different mean temperatures
Source: eLife. 2022 Feb 15;11:e72861. doi: 10.7554/eLife.72861 (PMC8846586; doi:10.7554/eLife.72861)
Supplement: Transparent reporting form [file elife-72861-transrepform1.docx]

***eLife’s* transparent reporting form**

We encourage authors to provide detailed information *within their submission* to facilitate the interpretation and replication of experiments. Authors can upload supporting documentation to indicate the use of appropriate reporting guidelines for health-related research (see [EQUATOR Network](http://www.equator-network.org/%20)), life science research (see the [BioSharing Information Resource](https://biosharing.org/)), or the [ARRIVE guidelines](http://www.plosbiology.org/article/info:doi/10.1371/journal.pbio.1000412) for reporting work involving animal research. Where applicable, authors should refer to any relevant reporting standards documents in this form.

If you have any questions, please consult our Journal Policies and/or contact us: [editorial@elifesciences.org](mailto:editorial@elifesciences.org).

**Sample-size estimation**

- You should state whether an appropriate sample size was computed when the study was being designed
- You should state the statistical method of sample size computation and any required assumptions
- If no explicit power analysis was used, you should describe how you decided what sample (replicate) size (number) to use

Please outline where this information can be found within the submission (e.g., sections or figure legends), or explain why this information doesn’t apply to your submission:

Prior experience with the *Daphnia* host and data from multiple experiments on the thermal tolerance of the used pathogen, *Ordospora* (Kirk et al., 2018, 2019, 2020), were used to determine the number of replicates and temperature treatments (that is, the total sample size). As *Daphnia* can show increased mortality in variable environments (Régnière et al., 2012), which could decrease the number of host available for pathogen measurements, the number of replicates in each temperature treatment slightly increased (from 12 to 18) for the treatments that experienced thermal variation. This information can be found on lines 323-329 and figure 1 in the manuscript.

**Replicates**

- You should report how often each experiment was performed
- You should include a definition of biological versus technical replication
- The data obtained should be provided and sufficient information should be provided to indicate the number of independent biological and/or technical replicates
- If you encountered any outliers, you should describe how these were handled
- Criteria for exclusion/inclusion of data should be clearly stated
- High-throughput sequence data should be uploaded before submission, with a private link for reviewers provided (these are available from both GEO and ArrayExpress)

Please outline where this information can be found within the submission (e.g., sections or figure legends), or explain why this information doesn’t apply to your submission:

The experiment was conducted once, with high levels of replication and multiple treatments. Considering that we use life animals for our experiments without repeated measurements and destructive sampling we believe that the definition of biological versus technical replication is not needed as it should be clear from the context of the manuscript that we are referring to biological replication. As animals that die early cannot be accurately scored for infection (that is infection has not established at a level where it is easily detectable by microscopy) any individuals that died before day 11 were excluded from the analysis. In addition, to prevent potentially confounding effects of animals that died early (where the parasite had less time to grow) as having lower spore burden, we included only animals from the last day of the experiment in the analysis for spore burden. Any male *Daphnia* that were misidentified as female at the start of the experiment were also excluded (the sex can be accurately observed while dissecting the *Daphnia* for parasite identification). All remaining data were included in the analysis. This information can be found in the manuscript on lines 361-366 and datafiles are available on <https://github.com/charlyknz/HostParasite.git>.

**Statistical reporting**

- Statistical analysis methods should be described and justified
- Raw data should be presented in figures whenever informative to do so (typically when N per group is less than 10)
- For each experiment, you should identify the statistical tests used, exact values of N, definitions of center, methods of multiple test correction, and dispersion and precision measures (e.g., mean, median, SD, SEM, confidence intervals; and, for the major substantive results, a measure of effect size (e.g., Pearson's r, Cohen's d)
- Report exact p-values wherever possible alongside the summary statistics and 95% confidence intervals. These should be reported for all key questions and not only when the p-value is less than 0.05.

Please outline where this information can be found within the submission (e.g., sections or figure legends), or explain why this information doesn’t apply to your submission:

Statistical methods are described and justified on lines 369-390 and additional information is provided in supplemental table S1. We fitted a beta function in a Bayesian framework. The parameters of this function have clear biological meaning and have been shown to fit well to thermal performance curves. Moreover, given this model system has been used in several climate change experiments a Bayesian approach allows us take advantage of these previous experiments and use informed priors. We do not display raw data as our samples size was over 10. Instead, we display the mean and the 95% confidence intervals for each of the different temperatures within each temperature regime. As we use Bayesian statistics, we do not report P-values but provide the 95% confidence intervals of the parameter estimates of the beta function.

(For large datasets, or papers with a very large number of statistical tests, you may upload a single table file with tests, Ns, etc., with reference to sections in the manuscript.)

**Group allocation**

- Indicate how samples were allocated into experimental groups (in the case of clinical studies, please specify allocation to treatment method); if randomization was used, please also state if restricted randomization was applied
- Indicate if masking was used during group allocation, data collection and/or data analysis

Please outline where this information can be found within the submission (e.g., sections or figure legends), or explain why this information doesn’t apply to your submission:

Animals were distributed randomly over the different temperature regimes and their individual temperature treatments (line 338). As we had to keep track of individual microcosms over the course of the experiment to ensure they received the correct temperature treatment it was not possible to fully mask the identity of the samples during the experiment. However, for observations of infection status and parasite burden, which requires two people (one to process the sample and one to determine infection status), the person who did the observation was unaware of the identity of the sample (line 360).

**Additional data files (“source data”)**

- We encourage you to upload relevant additional data files, such as numerical data that are represented as a graph in a figure, or as a summary table
- Where provided, these should be in the most useful format, and they can be uploaded as “Source data” files linked to a main figure or table
- Include model definition files including the full list of parameters used
- Include code used for data analysis (e.g., R, MatLab)
- Avoid stating that data files are “available upon request”

Please indicate the figures or tables for which source data files have been provided:

R code and datasets for host fecundity, parasite infectivity and parasite burden are available on GitHub <https://github.com/charlyknz/HostParasite.git>. Estimates of the Beta Equation parameters, their sample size and their 95% confidence intervals are provided in the supplementary tables S2, S3 and S4.
